# Distress, Work Satisfaction, and Work Ability are Mediators of the Relation Between Psychosocial Working Conditions and Mental Health-Related Long-Term Sickness Absence

**DOI:** 10.1007/s10926-020-09931-w

**Published:** 2020-10-19

**Authors:** Marieke F. A. van Hoffen, Judith J. M. Rijnhart, Giny Norder, Lisanne J. E. Labuschagne, Jos W. R. Twisk

**Affiliations:** 1 Department of Research and Development, Human Total Care, Utrecht, The Netherlands; 2 Department of Epidemiology and Biostatistics, VU University, VU University Medical Center, Amsterdam, The Netherlands; 3 Department of Otorhinolaryngology and Head and Neck Surgery, Erasmus University Medical Center, Rotterdam, The Netherlands; 4 The Generation R Study Group, Erasmus University Medical Center, Rotterdam, The Netherlands; 5 HumanCapitalCare, Laan van Nieuw Oost-Indië 133-G, 2593 BM Den Haag, The Netherlands

**Keywords:** Health surveys, Mental health, Mediation analysis, Psychosocial working, Conditions, Sick leave

## Abstract

*Purpose* This study investigated the effects of psychosocial working conditions on mental health-related long-term sickness absence and whether distress, work satisfaction, burnout, engagement, and work ability mediated the associations between psychosocial working conditions and mental health-related long-term sickness absence. *Methods* This cohort study included 53,833 non-sick listed workers who participated in occupational health surveys between 2010 and 2013. The effects of the individual psychosocial working conditions on mental long-term sickness absence were analyzed using univariable and multivariable logistic regression analyses. Mediation analyses were performed to examine the mediating role of distress, burnout, work satisfaction, engagement, and work ability between psychosocial working conditions and mental long-term sickness absence. The mediation analyses were performed using structural equation modeling. *Results* Role clarity, cognitive demands, emotional demands, work variety, learning opportunities, and co-worker support were related to mental health-related long-term sickness absence after adjustment for other working conditions. The relationship between emotional demands and mental health-related long-term sickness absence was the strongest, OR 1.304 (p < 0.001, 95% CI 1.135 to 1.498). The relation between psychosocial working conditions and mental health-related long-term sickness absence was mediated by distress, burnout, work satisfaction, engagement, and work ability. Distress was the most important mediator between psychosocial working conditions and mental health-related long-term sickness absence. *Conclusions* Psychosocial working conditions are related to mental health-related long-term sickness absence. After correction for other working conditions, the association between emotional demands and mental health-related long-term sickness absence was the strongest. Psychosocial working conditions are indirectly related to mental health-related long-term sickness absence through mediation by distress, work satisfaction, and work ability.

## Introduction

Mental health problems are the most important and increasing cause of long-term sickness absence (LTSA) of the workforce. The Organization of Economic Cooperation and Development (OECD) reported in 2015 that 30 to 40% of the sickness absence and work disability cases in western societies were related to mental illness [1]. LTSA disengages workers from the workplace and the probability of resuming work decreases with increasing LTSA duration. LTSA due to a mental illness has a median duration of 231 days (data of HumanTotalCare, The Netherlands 2018). To prevent mental health-related LTSA, it is important to identify the causal mechanisms underlying mental health-related LTSA.

There is evidence that psychosocial working conditions are associated with mental health-related LTSA [2, 3]. The psychosocial working conditions are formed by a combination of job demands (e.g. work pace, cognitive demands, emotional demands, work-family interference) and job resources (e.g. role clarity, variety in work, learning opportunities, supervisor support, and co-worker support). Our study is based on the Job Demands-Resources (JD-R) model*,* which is one of the theoretical models looking at the relationship between psychosocial work factors and mental health and sickness absence. The JD-R model describes that adverse psychosocial working conditions lead to emotional exhaustion and burnout if the efforts to meet job demands are too high or if there is insufficient time to recover from the demands, i.e. the exhaustion process [4]. On the other hand, high job resources enable coping with job demands, to achieve goals, stimulate personal growth and lead to work satisfaction. i.e. the motivational process [5–7].

Previous research has shown that adverse psychosocial working conditions lead to distress [8–13] and that sustained distress leads to mental health-related LTSA [14, 15]. It is therefore expected that distress will mediate the relation between psychosocial working conditions and mental health-related LTSA.

There is also evidence of an association between psychosocial working conditions and burnout. Schaufeli et al. [16] found a relation between high work pace, high emotional demands, high work family interference and higher burnout. Fagerlind Ståhl et al. [17] showed that high demands such as work pace, workload and conflicting demands at work were associated with greater risk of burnout. Burnout in turn is associated with a higher risk of sickness absence [18].

Several studies described associations between psychosocial working conditions and work satisfaction. High work pace was found to be related to low job satisfaction [13, 16], whereas de Jonge [19] showed a relation between high emotional demands and low work satisfaction. Furthermore, low work satisfaction was associated with higher sickness absence by Laaksonen et al. [20].

There is consistent evidence of an association between challenging job demands combined with high job resources and high engagement [4, 21]. Low engagement was found to be associated with high sickness absence [22].

Finally, work ability, is also expected to be a mediator between psychosocial working conditions and mental health-related LTSA since work ability includes a component related to mental capability to perform at work [23]. Previous research showed that psychosocial working conditions were related to work ability [24, 25] and work ability was found to be related to mental health-related LTSA [26].

**Fig. 1:**
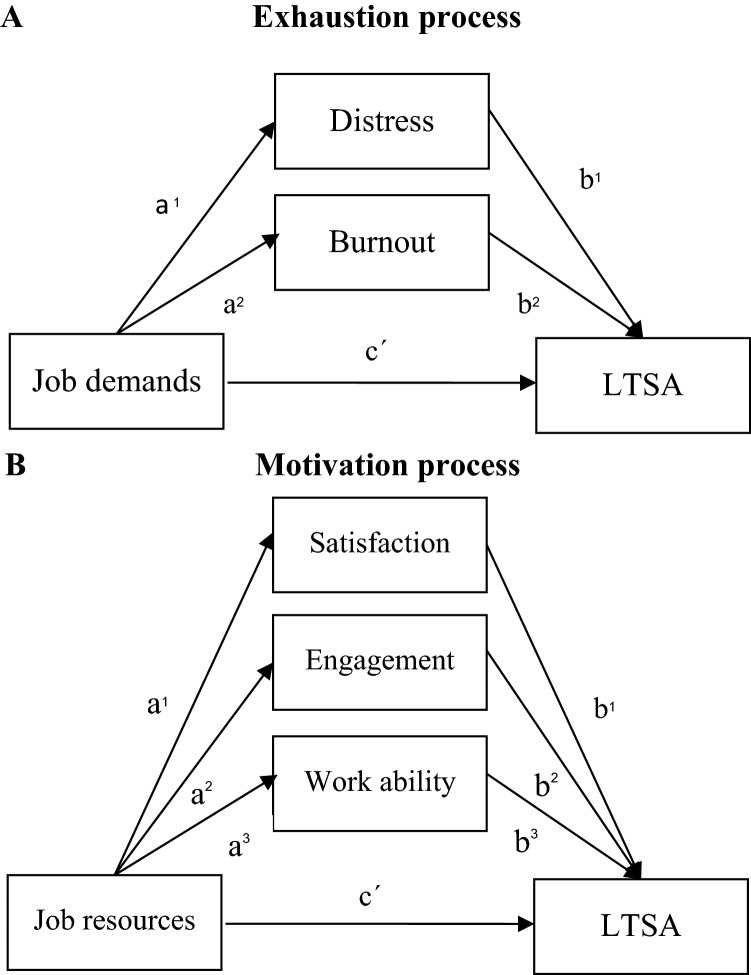
Multiple mediator models. The model in panel **A** was corrected for job resources. The model in panel **B** was adjusted for job demands

The aforementioned associations have only been investigated individually and mostly without mental health-related LTSA data hence a cohesive understanding of the causal mechanisms in the relation between psychosocial working conditions and mental health-related LTSA is still lacking. The current study therefore investigates these associations using mediation analyses. Figure 1 shows our hypothesized mediation model. In Fig. 1c’ reflects the direct paths and the indirect paths are reflected by the products of a and b.

The aim of this study was to investigate the total, direct, and indirect effect of psychosocial working conditions on mental health-related LTSA. We investigated if distress, burnout, work satisfaction, engagement, and work ability mediated the associations between psychosocial working conditions and mental health-related LTSA.

## Methods

### Study Population and Design

For this study, we used the data of 53,833 workers who participated in occupational health surveys in The Netherlands between 2010 and 2013. According to the Dutch Labor Law, companies have to enable their employees to participate in a voluntary occupational health survey once every four years. Occupational health surveys are conducted by occupational health services (OHS) and consist of an online occupational health questionnaire. The questionnaire commonly addresses physical and mental workload, psychosocial work environment, working conditions, and health complaints. The study was set up as a prospective cohort study with the occupational health survey measured at baseline and sickness absence recorded in the year following the occupational health survey at follow-up. Participants with more than 25% missing responses or on sickness absence at baseline were excluded from our study, leaving the data of 31,884 non-sick-listed (57%) participants for complete case analyses. Participants with complete data did not differ from excluded participants in age, gender, education, and years employed at the company. Missing data were therefore assumed to be completely at random. The Medical Ethics Committee of the University Medical Center Groningen reviewed the study and granted ethical approval.

### Outcome: Long-Term Sickness Absence (LTSA)

Sickness absence was defined as a temporary paid leave from work due to any (i.e., work-related as well as non-work-related) injury or illness, and was recorded from the first to the last sickness absence day in an occupational health service (OHS) register. In The Netherlands, sickness absence is medically certified by an occupational physician (OP) within 42 days of reporting sick. Therefore, LTSA was defined as sickness absence lasting 42 days or longer.

Based on a consultation with a sick-listed worker, the OP records a diagnostic code derived from the 10th International Classification of Diseases (ICD-10) in the OHS register. Mental health-related LTSA was defined as LTSA with diagnostic codes of the ICD-10 chapter V (Mental and Behavioral Disorders). Mental health-related LTSA during 1-year follow-up was used as the dichotomous outcome variable. The exposed group was the group with mental LTSA, while the workers without sickness absence lasting 42 days or longer or any other diagnosis comprised the reference group.

### Independent Variables

#### Psychosocial Working Conditions

The job demands, work pace (5 items, Cronbach’s α = 0.87), cognitive demands (5 items, α = 0.82), emotional demands (3 items, α = 0.80), and job resources, variety in work (6 items; α = 0.86), role clarity (5 items; α = 0.85), learning opportunities (4 items; α = 0.87), supervisor support (3 items; α = 0.90), and co-worker support (3 items; α = 0.88), were measured with the Questionnaire on the Experience and Evaluation of Work [27]. Survey participants responded on a five-point frequency scale ranging from 1 (i.e. ‘never’) to 5 (i.e. ‘always’) and item scores were summed to a total subscale score, which was then divided by the number of items of that subscale. Consequently, all psychosocial working characteristics consisted of a score ranging between 1 (i.e. low) and 5 (i.e. high).

The job demand work – family interference was assessed with 7 items (e.g., “How often does your job interfere with responsibilities at home?”, “How often does your job prevent you from spending time with family and friends?” α = 0.88). Responses were given on 5-point frequency scales ranging from ‘never’ (i.e. 1) to ‘always’ (i.e. 5); item scores were summed and averaged so that work family interference ranged between 1 (i.e. low) and 5 (i.e. high).

#### Mediators

Distress was measured with the Four-Dimensional Symptom Questionnaire (4DSQ), which was included in the occupational health survey questionnaire [28, 29]. The distress scale consisted of 16 items addressing symptoms elicited by stressors or the efforts to maintain psychosocial functioning, e.g., worry, irritability, tension, listlessness, poor concentration, sleeping problems, and demoralization [28, 29]. Survey participants were asked if they had experienced these symptoms in the past week, ‘no’ (i.e. 0), ‘sometimes’ (i.e. 1), ‘regularly’ (i.e. 2), ‘often’ (i.e. 2), or ‘very often/constantly’ (i.e. 2). Item scores were summed (score range 0–32; Cronbach’s α = 0.94), so that higher scores reflected higher levels of distress. Terluin et al. [30] defined scores ≤ 10 as low, 11–20 as moderate, and > 20 as high distress.

Burnout was measured with the 15-item Dutch version of the Maslach Burnout Inventory – General Scale [31]. Items were scored on a 6-point frequency scale, summed, and averaged into a burnout score between 0 (i.e. low) and 6 (i.e. high).

Work satisfaction was measured with 6 items (α = 0.87) about pleasure in work (e.g., “I am pleased to start my day’s work”, “I find my work stimulating”, “I enjoy my work”). Responses were given on a 5-point frequency scale ranging from ‘never’ (i.e. 1) to ‘always’ (i.e. 5). Items scores were summed and averaged, so that work satisfaction ranged between 1 (i.e. low) and 5 (i.e. high).

Work engagement was measured with a 9-item short form of the Utrecht Work Engagement Scale [32]. The items were scored on a 6-point frequency scale ranging from ‘never’ (= 0), ‘scarcely’ (= 1), ‘sometimes’ (= 2), ‘regularly’ (= 3), ‘often’ (= 4), ‘very often’ (= 5), and ‘always’ (= 6). The item scores were summed and averaged to a work engagement score between 0 (i.e. low) and 6 (i.e. high).

Work ability was measured with a shortened version of the Work Ability Index (WAI) covering items on current work ability compared with lifetime best work ability in relation to the (physical and mental) demands of work, number of physician-diagnosed diseases, impaired work performance due to illness, sickness absence in the past 12 months, expected work ability in the forthcoming two years, and mental resources [33]. The item scores were summed to a total work ability score ranging from 7 (i.e. poor) to 49 (i.e. excellent).

### Statistical Analyses

To analyze the effect of psychosocial working conditions on mental health-related LTSA and whether distress, burnout, work satisfaction, engagement, and work ability mediated these associations, three types of statistical analyses were performed. First the total effects of job demands on mental health-related LTSA and the effect of job resources on mental health-related LTSA were assessed using multivariable logistic regression analyses with and without confounders. Second, to assess the mutual influences of the psychosocial working conditions, i.e., job demands and job resources, a multivariable logistic regression model was used in which the relationships between all job demands and job resources on the one hand and mental health-related LTSA on the other hand were analyzed simultaneously.

Third, multiple mediator models were used to assess the mediating role of distress and burnout in the associations between job demands and mental health-related LTSA, and to assess the mediating rom of work satisfaction, engagement, and work ability in the associations between job resources and mental health-related LTSA. The mediation analyses were performed using structural equation modeling (SEM) [34]. We estimated the effects of psychosocial working conditions on the mediators using linear regression (*a* paths), and the effects of the mediators on mental health-related LTSA (*b* paths) and the effects of psychosocial working conditions on mental LTSA (*c* paths) using logistic regression. Based on these pathways, the indirect effect of each psychosocial working condition on mental LTSA via a mediator was calculated as the product of the *a* and *b* path [35]. For each indirect effect a 95% percentile bootstrap confidence interval was calculated based on 1,000 bootstrap resamples [36]. The multiple mediator model based on job demands was adjusted for job resources, and the multiple mediator model based on job resources was adjusted for job demands. All analyses were performed in STATA 14 (StataCorp LP, College Station, TX, USA). Before analysis, all psychosocial working conditions were standardized and thus directly comparable.

## Results

The 31,884 (59%) non-sick-listed occupational health survey participants with complete data were more often married women with higher education, working for a shorter time in their present job, and with more hours per week as compared to those excluded because of missing data, although the differences were small.

The survey participants (77% men) had a mean age of 45.2 years (standard deviation [SD] = 10.1) and were working an average of 38.4 h per week (SD = 7.7) for 14.4 years (SD = 11.5). Of all participants, 18% had a lower education, 44% a medium education, and 38% were highly educated. The sectors they worked in were agriculture (3%), industry (71%), commercial services (14%), and public services (12%) (Table 1).

**Table 1:** Population characteristics (N = 53,833)

	Complete cases analysis (n = 31,884)				Excluded cases^a^ (n = 21,949)				Analysis
	Mean	SD^b^	n	%	Mean	SD	n	%	
Age	45.2	10.1			44.7	10.9			P < 0.01
Gender									P < 0.01
Men			24,499	77			17,539	80	
Women			7385	23			4289	20	
Missing			–				121		
Marital status									P < 0.01
Single			3233	10			2710	12	
Relationship, but living apart			2600	8			1864	9	
Living together/married			25,373	80			15,708	72	
Other			678	2			1043	5	
Missing			–				624		
Care for children at home									P = 0.21
No			13.069	41			7234	40	
Yes			18,815	59			10,714	60	
Missing			–				4,001		
Education									P < 0.01
Low			5284	17			4143	19	
Medium			13,660	43			9925	46	
High			12,940	40			7405	34	
Missing			–				476		
Economic sector									P < 0.01
Agriculture			893	3			198	1	
Industry			22,637	71			15,827	72	
Commercial services			4464	14			2984	14	
Public services			3890	12			2940	13	
Missing			–				–		
Years employed at company	14.4	11.5			17.0	12.5			P < 0.01
Years in present job	8.4	8.3			9.0	9.1			P < 0.01
Work hours a week	38.4	7.7			37.6	7.3			P < 0.01
Social support family/friends (range 1–5)	3.6	1.0			3.5	1.0			P < 0.01
Prior mental LTSA^c^									P = 0.23
Yes			476	2			359	2	
No			31,408	98			21,603	98	
Missing			–				–		
Psychosocial work factors (range 1–5)									
Work pace	2.8	0.7			2.7	0.8			P < 0.01
Cognitive demands	3.6	0.7			3.5	0.7			P < 0.01
Emotional demands	1.7	0.6			1.7	0.6			P < 0.01
Variety in work	3.6	0.8			3.6	0.8			P < 0.01
Role clarity	4.0	0.7			4.0	0.7			P = 0.06
Learning opportunities	3.1	1.0			3.0	1.0			P < 0.01
Supervisor support	3.6	1.0			3.6	1.0			P < 0.01
Co-worker support	3.9	0.8			3.9	0.8			P < 0.01
Organizational commitment	3.2	0.7			3.1	0.7			P < 0.01
Work–family interference (range 1–5)	1.7	0.6			1.6	0.6			P < 0.01
Intrinsic work motivation (1–7)	5.9	1.0			5.9	1.0			P < 0.01
Work satisfaction (range 1–5)	3.9	0.8			3.9	0.8			P < 0.01
Work ability (7–49)	42.2	4.2			42.2	4.2			P = 0.20
Work engagement (range 0–6)	3.8	1.1			3.7	1.1			P < 0.01
Burnout (range 0–6)	2.4	0.5			2.4	0.5			P = 0.48
Distress									P < 0.01
Low			22,740	71			15,254	73	
Medium			6664	21			4179	20	
High			2480	8			1463	7	
Missing			–				1053		

^a^Excluded because of baseline sickness absence or missing responses

^b^Standard deviation

^c^Long-term sickness absence due to mental disorders in the 12 months before baseline

Of the 31,884 occupational health survey participants with complete data, 466 (1.5%) had mental LTSA during 1-year follow-up.

Table 2 shows the results of the univariable and multivariable logistic regression analyses, in which the psychosocial working conditions were related to mental health-related LTSA. In the univariable analyses the job demands work pace, emotional demands, and work-family interference, and the job resources role clarity, learning opportunities, supervisor support, and co-worker support were significantly associated with mental health-related LTSA. However, after correction for gender, marital status, care for children at home, education, age, years employed at company, work hours per week, support from family and friends, and prior long-term sickness absence due to mental complaints, only the associations of emotional demands, work-family interference, learning opportunities, and co-worker support remained significant. The relationship between emotional demands and mental health-related LTSA was the strongest after correction for other working conditions, OR 1.304 (p < 0.001, 95% CI 1.135 to 1.498).

**Table 2:** Unadjusted and adjusted relationships between psychosocial working conditions and mental LTSA

	Without confounders						With confounders^a^		
Psychosocial working condition	OR	95% CI ^b^	P-value	OR	95% CI	P-value	OR	95% CI	P-value
Job demands							Adjusted for job resources		
Work pace	1.141	1.005 to 1.297	0.042	1.133	0.997 to 1.288	0.055	1.088	0.958 to 1.236	0.195
Cognitive demands	0.898	0.775 to 1.039	0.148	0.957	0.828 to 1.106	0.549	1.045	0.902 to 1.210	0.559
Emotional demands	1.411	1.229 to 1.620	0.000	1.367	1.191 to 1.570	0.000	1.304	1.135 to 1.498	0.000
Work-family interference	1.204	1.036 to 1.398	0.015	1.303	1.117 to 1.520	0.001	1.120	1.027 to 1.402	0.022
Job resources							Adjusted for job demands		
Role clarity	0.847	0.739 to 0.971	0.017	0.894	0.775 to 1.031	0.123	0.937	0.812 to 1.083	0.380
Variety in work	0.983	0.859 to 1.126	0.809	1.110	0.962 to 1.281	0.152	1.009	0.872 to 1.169	0.900
Learning opportunities	0.852	0.750 to 0.969	0.015	0.838	0.735 to 0.956	0.008	0.846	0.742 to 0.964	0.012
Supervisor support	0.888	0.794 to 0.994	0.038	0.863	0.772 to 0.966	0.010	0.897	0.802 to 1.003	0.056
Co-worker support	0.797	0.709 to 0.896	0.000	0.833	0.739 to 0.938	0.003	0.867	0.770 to 0.976	0.000

^a^Confounders: gender, marital status, care for children at home, education, age, years employed at company, work hours a week, support from family and friends, prior sickness long term sickness absence due to mental complaints

^b^CI = confidence interval

### Mediation Analyses

#### Job Demands

Table 3 shows the direct effects of the job demands and job resources on mental health-related LTSA, the indirect effects of job demands through distress and burnout on mental health-related LTSA, and of job resources through work satisfaction, engagement, and work ability on mental health-related LTSA. Distress was the most important mediator between job demands and mental health-related LTSA and mediated the effect of work-family interference (OR 1.213; 95% CI 1.167–1.261), emotional demands (OR 1.151; 95% CI 1.119–1.184), and work pace (OR 1.056; 95% CI 1.043–1.070) on mental health-related LTSA. Emotional demands had the highest remaining direct effect on mental health-related LTSA (OR 1.144; 95% CI 0.994–1.317), but the effect was not significant.

**Table 3:** Mediation effects of distress, burnout, work satisfaction, engagement, and work ability in the relationship between job demands and resources and mental LTSA

Job demands	Exhaustion path ^b^ Mediator distress					Exhaustion path with job resources as confounders ^c^ Mediator dIstress				
	OR Direct effect	95% CI ^a^	*p*	OR Indirect effect	95% CI	OR Direct effect	95% CI ^a^	*p*	OR Indirect effect	95% CI ^a^
Work pace	1.051	0.926 to 1.195	0.430	1.056	1.043 to 1.070	1.037	0.913 to 1.178	0.574	1.047	1.035 to 1.060
Cognitive demands	0.952	0.825 to 1.099	0.499	1.009	0.995 to 1.010	0.977	0.843 to 1.132	0.752	1.051	1.039 to 1.064
Emotional demands	1.144	0.994 to 1.317	0.060	1.151	1.119 to 1.184	1.127	0.979 to 1,298	0.096	1.137	1.106 to 1.168
Work-family interference	1.014	0.864 to 1.190	0.865	1.213	1.167 to 1.261	0.998	0.850 to 1.172	0.985	1.170	1.131 to 1.209
Job demands	Mediator burnout					Mediator burnout				
Work pace	1.051	0.926 to 1.195	0.430	1.016	0.994 to 1.032	1.037	0.913 to 1.178	0.574	1.009	0.993 to 1.024
Cognitive demands	0.952	0.825 to 1.099	0.499	1.009	0.999 to 1.017	0.977	0.843 to 1.132	0.752	1.010	0.993 to 1.025
Emotional demands	1.144	0.994 to 1.317	0.060	1.023	0.999 to 1.047	1.127	0.979 to 1,298	0.096	1.013	0.990 to 1.035
Work-family interference	1.014	0.864 to 1.190	0.865	1.031	0.999 to 1.062	0.998	0.850 to 1.172	0.985	1.016	0.989 to 1.042

Job resources	Motivation path ^b^ Mediator work satisfaction					Motivation path with job demands as confounders ^d^ Mediator work satisfaction				
	OR Direct effect	95% CI ^a^	*p*	OR Indirect effect	95% CI	OR Direct effect	95% CI ^a^	*p*	OR Indirect effect	95% CI ^a^
Role clarity	1.018	0.879 to 1.178	0.811	0.956	0.932 to 0.984	1.033	0.892 to 1.197	0.665	0.966	0.943 to 0.993
Variety in work	1.231	1.061 to 1.427	0.006	0.954	0.929 to 0.984	1.133	0.973 to 1.320	0.108	0.957	0.929 to 0.992
Learning opportunities	0.923	0.807 to 1.726	0.244	0.959	0.936 to 0.985	0.920	0.804 to 1.052	0.224	0.966	0.942 to 0.993
Supervisor support	0.902	0.806 to 1.009	0.074	0.978	0.966 to 0.993	0.926	0.827 to 1.036	0.178	0.984	0.973 to 0.997
Co-worker support	0.870	0.772 to 0.980	0.022	0.986	0.978 to 0.995	0.894	0.793 to 1.007	0.065	0.991	0.983 to 0.998
	Mediator engagement					Mediator engagement				
Role clarity	1.018	0.879 to 1.178	0.811	0.985	0.953 to 1.021	1.033	0.892 to 1.197	0.665	0.982	0.950 to 1.016
Variety in work	1.231	1.061 to 1.427	0.006	0.980	0.936 to 1.029	1.133	0.973 to 1.320	0.108	0.974	0.929 to 1.023
Learning opportunities	0.923	0.807 to 1.726	0.244	0.987	0.957 to 1.019	0.920	0.804 to 1.052	0.224	0.983	0.954 to 1.015
Supervisor support	0.902	0.806 to 1.009	0.074	0.996	0.986 to 1.007	0.926	0.827 to 1.036	0.178	0.995	0.986 to 1.004
Co-worker support	0.870	0.772 to 0.980	0.022	0.996	0.988 to 1.005	0.894	0.793 to 1.007	0.065	0.995	0.987 to 1.004
	Mediator work ability					Mediator work ability				
Role clarity	1.018	0.879 to 1.178	0.811	0.935	0.914 to 0.956	1.033	0.892 to 1.197	0.665	0.954	0.935 to 0.974
Variety in work	1.231	1.061 to 1.427	0.006	0.966	0.954 to 0.977	1.133	0.973 to 1.320	0.108	0.963	0.947 to 0.979
Learning opportunities	0.923	0.807 to 1.726	0.244	0.971	0.961 to 0.981	0.920	0.804 to 1.052	0.224	0.977	0.967 to 0.987
Supervisor support	0.902	0.806 to 1.009	0.074	0.988	0.983 to 0.993	0.926	0.827 to 1.036	0.178	0.995	0.991 to 0.998
Co-worker support	0.870	0.772 to 0.980	0.022	0.981	0.973 to 0.987	0.894	0.793 to 1.007	0.065	0.989	0.983 to 0.994

^a^*CI* confidence interval

^b^With confounders gender, marital status, care for children at home, education, age, years employed at company, work hours a week, support from family and friends, and prior sickness long term sickness absence due to mental complaints

^c^Model with job demands and confounders gender, marital status, care for children at home, education, age, years employed at company, work hours a week, support from family and friends, prior sickness long term sickness absence due to mental complaints and job resources as confounders

^d^Model with job resources and confounders gender, marital status, care for children at home, education, age, years employed at company, work hours a week, support from family and friends, prior sickness long term sickness absence due to mental complaints, and job demands as confounders

Burnout was not a mediator in the association between job demands and mental health-related LTSA.

Adding job resources to the mediation analysis of distress and burnout on mental health-related LTSA had little effect.

#### Job Resources

Work satisfaction was a mediator of the relationship between role clarity (OR 0.956; 95% CI 0.932 to 0.984), variety in work (OR 0.954; 95% CI 0.929–0.984), learning opportunities (OR 0.959; 95% CI 0.936–0.985), supervisor support (OR 0.978; 95% CI 0.966–0.993), co-worker support (OR 0.986; 95% CI 0.978–0.995) and mental LTSA. Variety in work (OR 1.231; 95% CI 1.061–1.427) and co-worker support (OR 0.870; 95% CI 0.772–0.980) had a remaining direct effect on mental health-related LTSA.

Engagement was not a mediator between job resources and mental health-related LTSA.

Finally, work ability mediated the relationship between role clarity (OR 0.935; 95% CI 0.914–0.956), variety in work (OR 0.966; 95% CI 0.954–0.977), learning opportunities (OR 0.971; 95% CI 0.961–0.981), supervisor support (OR 0.988; 95% CI 0.983–0.993), and co-worker support (OR 981 95% CI 0.973–0.987) and mental health-related LTSA. Variety in work (OR 1.231; 95% CI1.061–1.427) and co-worker support (OR 0.870; 95% CI 0.772–0.980) had a remaining direct effect on mental health-related LTSA.

Adding job demands to the analysis of the mediational effect of work satisfaction, engagement, and work ability on mental health-related LTSA had little effect on the outcome.

## Discussion

The present study investigated the direct and indirect effects of psychosocial working conditions on mental health-related LTSA. The job demands emotional demands and work-family interference, and the job resources learning opportunities, supervisor support, and co-worker support were associated with mental health-related LTSA. After correction for other job resources and confounders, the relationship between emotional demands and mental health-related LTSA was the strongest.

High emotional demands and high work-family interference were associated with higher mental health-related LTSA. High learning opportunities, high supervisor support, and high co-worker support were associated with lower mental health-related LTSA.

The literature on associations of job demands and job resources with mental health-related LTSA is scarce and contradictory. Aronsson [37] reported that high emotional demands were associated with higher sickness absence. In a study by Slany et al. [3] that was done across European countries, i.e. with different working populations and settings, the researchers were able to find clear associations for several psychosocial work factors (such as learning opportunities and social support), but not for emotional demands. In contrast to our findings, Janssen et al. [38] found no association between supervisor support and mental health-related LTSA. In addition Munir et al. [39] found no effect of co-worker support on mental health-related LTSA. Our finding that higher work-family interference was associated with higher mental health-related LTSA is in line with earlier studies [40, 41]

Potentially, study design (cross-sectional vs. prospective) could influence the differences in relationships found. Another explanation could be that the surveys used different questionnaires in various studies. Furthermore, an explanation for any discrepancy could be that associations of job demands and resources vary across working populations and workplace settings [42].

In addition, we investigated the potential mediation of several factors in the relationship between job demands and job resources and mental health-related LTSA. The associations between psychosocial working conditions and mental health-related LTSA were mediated by distress, work satisfaction, and work ability. Distress mediated the associations between the investigated job demands and mental health-related LTSA. This confirmed the hypothesis that high job demands lead to distress, through the exhaustion process [4], which in turn leads to mental health-related LTSA. In this study we found that high emotional demands and high work-family interference lead to high mental health-related LTSA, which effects were mediated by distress. Previous research has shown that adverse psychosocial working conditions lead to distress {8–13] and burnout {16.17]. In turn, sustained distress [14, 15] and burnout ( [18] lead to mental health-related LTSA. The finding that distress mediated the relations between psychosocial working conditions and mental health-related LTSA was therefore expected, but to our knowledge not examined in research before. The mediational effect of burnout on the associations between job demands and mental health-related LTSA was also expected, yet not confirmed in our study.

Job resources are described to buffer the effect of job demands on mental LTSA [6]. However, adding job resources to our analysis of the mediational effect of distress and burnout on mental health-related LTSA had little effect on the outcome.

In this study we found that work satisfaction and work ability mediated the effect of role clarity, learning opportunities, and variety in work on mental health-related LTSA. We found that the effects of all analyzed job resources (role clarity, variety in work, learning opportunities, supervisor support, and co-worker support) on mental health-related LTSA were mediated by work satisfaction as well as work ability. This indicates that work satisfaction and work ability play an important role in the motivational process through which psychosocial working conditions operate. The role of work satisfaction and engagement in the motivational process has been described earlier [4, 21]. Previous research found an association between engagement and work ability [22, 43]. The current study confirmed the suspected role of work ability in the motivational process. Although the mediational role of work satisfaction and work ability in the association between psychosocial working conditions and mental health-related LTSA could be expected, it was to our knowledge not investigated before in a mediation analysis. The mediational effect of engagement on the relation between job resources and mental health-related mental LTSA was also expected, yet not confirmed in our study. Although not described in literature, we wanted to investigate if job demands do buffer the effect of job resource on mental LTSA. Adding job demands to our analysis of the mediational effect of work satisfaction, engagement, and work ability had little effect on the outcome.

### Strengths and Limitations

The large study population, prospective study design, and the use of recorded OP-certified mental health-related LTSA were strengths of the study. It is one of the few studies looking into explanatory mechanisms of the relationship between psychosocial work characteristics and mental health-related LTSA. Another strength of this study was that multiple mediators were investigated. Although large, the study population was not representative of the Dutch workforce, since industry and commercial business sectors were overrepresented and agriculture and public services were underrepresented. Therefore the results of our study cannot be applied to all sectors and we advise to repeat this study in other economic sectors.

Forty-three percent of the participants were excluded because of missing data. However, we assumed the data to be missing completely at random following the comparison we made based on three characteristics, which justified the use of complete case analysis.

Furthermore, the psychosocial working conditions in the current study were measured with a questionnaire and therefore reflected the worker’s subjective perception of the psychosocial working conditions rather than an objective one. Rehkopf et al. [44] reported that external measures of psychosocial working conditions were more strongly associated with higher sickness absence compared with self-assessed measures. It would be interesting to repeat our study with externally measured psychosocial working conditions.

The association between the psychosocial working conditions and mediation factors may have been a result of reversed causality, because they were measured at the same time. We theorized that job demands and resources had effects on the mediators. In reality, these effects are more complex and can be reciprocal, which provides directions for future research. For example, future research could be conducted in which the reciprocal effects of psychosocial working conditions, distress, burnout, work satisfaction, engagement, work ability, and mental health LTSA are investigated based on longitudinal data using cross-lagged panel models [35, 45, 46].

### Practical Implications

The guideline of the Netherlands Society of Occupational Medicine [47] states that occupational health physicians should explore the causes of mental complaints during consultations. According to our findings, low learning opportunities, low co-worker and supervisor support, high emotional demands, and high work-family interference are associated with mental health-related LTSA. We therefore advise that during occupational health consultations, particular interest is paid to these psychosocial working conditions. In addition, distress, job satisfaction, and work ability are advised to be investigated, since they seem to play a mediational role in the exhaustion and motivation processes. In order to prevent mental health-related LTSA, companies are therefore advised to take action to enhance learning opportunities, supervisor support, and co-worker support. These psychosocial working conditions together with emotional demands, work-family interference, distress, work satisfaction, and work ability are advised to be measured periodically in occupational health surveys. Employers could also train supervisors to recognize early signs of distress, dissatisfaction, and low work ability in their employees. Previous studies showed that preventive consultations with workers at risk of mental illness reduced the frequency and duration of mental health-related LTSA [48, 49]. Workers at risk of mental health-related LTSA can be invited for a consultation with an occupational physician or nurse and, if necessary, be referred to a psychologist to prevent them from experiencing mental health-related LTSA [50].

Since the effect of emotional demands on mental LTSA was found to be the strongest, it is important to teach workers by means of a preventive training to regulate their emotions on a daily basis [51]. There is increasing evidence that work breaks improve mental health of employees especially in prolonged high job demands [52–54]. Employers are advised to give workers time to recover during work in jobs with high emotional demands.

To our knowledge the mediational role of work ability has not been described before. We advise to repeat this study in other populations, especially in populations with more women such as healthcare and education.

## Conclusion

Psychosocial working conditions are related to mental health-related LTSA. After correction for other working conditions, the association between emotional demands and mental health-related LTSA was the strongest. Psychosocial working conditions are indirectly related to mental health-related LTSA by mediation of distress, work satisfaction, and work ability.
